# Association analysis of suicide risk assessed with Mini International Neuropsychiatric Interviews’ Suicidality Module in adolescents with non suicidal self injury disorder

**DOI:** 10.3389/fpsyt.2025.1546039

**Published:** 2025-02-10

**Authors:** Shuo Geng, Wen Zhang, Xiang Gao, Lele Qu, Xueping Zheng, Jian Sun, Mingdong Xu, Hua Lin, Xueyu Jia, Xu Zhang

**Affiliations:** ^1^ Qingdao Medical College, Qingdao University, Qingdao, Shandong, China; ^2^ Department of Psychiatry, The Affiliated Hospital of Qingdao University, Qingdao, Shandong, China; ^3^ School of Foreign Language Education, Qingdao University, Qingdao, Shandong, China; ^4^ Department of Allergy, The Affiliated Hospital of Qingdao University, Qingdao, Shandong, China; ^5^ Art College, Qingdao University of Science and Technology, Qingdao, Shandong, China; ^6^ The Geriatric Department, The Affiliated Hospital of Qingdao University, Qingdao, Shandong, China; ^7^ School of Stomatology of Qingdao University, The Affiliated Hospital of Qingdao University, Qingdao, Shandong, China; ^8^ Department of Clinical Nutrition, Qilu Hospital (Qingdao), Cheeloo College of Medicine, Shandong University, Qingdao, Shandong, China

**Keywords:** non suicidal self injury disorder, suicide risk, depression, anxiety, network analysis, adolescent

## Abstract

**Objective:**

Many adolescents with non suicidal self injury disorder have suicidal ideation. However, the specific characteristics of adolescents with NSSI-D that contribute to high suicide risk remain unclear. This study observes the association between depressive and anxiety symptoms and suicide risk among adolescents with non suicidal self injury disorder, and explores the mechanism underlying the high risk of suicide in this population.

**Method:**

Adolescents with non suicidal self injury disorder and their parents from a psychiatric outpatient clinic were selected to conduct paper questionnaires to measure their sociodemographic conditions. The Mini International Neuropsychiatric Interviews’ Suicidality Module(MINISM) was used to assess suicide risk of participants. The Self-Rating Depression Scale(SDS), Self-Rating Anxiety Scale(SAS), and Piers-Harris Children’s Self-Concept Scale(PHCSS) were used to measure depressive and anxiety symptoms, and self-concept of adolescents with non suicidal self injury disorder. According to the high suicide risk cutoff value of MINISM, the sample was divided into high suicide risk group and non-high suicide risk group. We performed descriptive and correlation statistical and network analysis to study the types of depressive and anxiety symptoms associated with suicide risk and the mechanism underlying suicide risk among non suicidal self injury disorder adolescents.

**Results:**

A total of 112 non suicidal self injury disorder adolescent participants were included in this study. Severe depressive symptoms(OR=8.205, 95%CI=3.454-19.490) and severe anxiety symptoms(OR=3.926, 95%CI=1.613-9.554) are associated with a high risk of suicide. The father’s college/university education(p<0.01) is associated with severe anxiety symptoms, and low self-concept(p<0.01) is associated with severe depressive symptoms. Network analysis suggests the centrality of anxiety symptoms and father’s education level.

**Conclusion:**

The results of statistical analysis suggest that severe depressive symptoms are related to the high risk of suicide (based on MINISM) in adolescents with NSSI-D statistical significantly, and anxiety symptoms and low self-concept are associated with depressive symptoms in NSSI-D adolescents. Interventions targeting anxiety symptoms in adolescents with NSSI-D may help reduce their suicide risk.

## 1 Introduction

Non suicidal self injury(NSSI) refers to active self-harm without suicidal intention, primarily to relieve negative emotions (1). In 5th edition of the Diagnostic and Statistical Manual of Mental Disorders(DSM-5), new criteria were proposed to distinguish NSSI, drawing significant attention from researchers and clinicians (2). Defining NSSI as a disorder is important, as it involves specific symptoms, including repeated engagement in low-risk self-harm behaviors such as hitting oneself or scratching the skin (3). Moreover, NSSI is linked to clinical and functional impairment (4). And it was strongly associated with increased risk of suicide, independent of other pre-existing psychiatric disorders (5). Given these factors, defining NSSI as a disorder rather than merely a behavior or symptom, and it can may provide valuable guidance and framework for clinical practice and scientific research.

A considerable number of studies have analyzed the etiology and related factors of NSSI-D, the underlying drivers remain incompletely understood. Many studies believe that a history of childhood abuse, family disharmony, and highly sensitive personality traits are the main risk factors (6). NSSI was more common in females ages 16-19, making adolescents a particular focus (7). The growing attention to NSSI-D in adolescents is largely due to its strong association with suicidal behaviors. Many studies reported that some adolescents with NSSI-D have suicidal ideation and suicide attempts (8, 9). However, the association between NSSI and suicide thoughts and attempts in low- and middle-income countries is often overlooked (10). Therefore, further investigation into suicide risk factors in adolescents with NSSI-D is needed.

NSSI-D often co-occurs with other mental health issues (11, 12), such as mood disorders. In general, adolescents with multiple mental health issues may have a worse prognosis (13). There is currently a lack of understanding of the association between prognostic risks and depressive or anxiety symptoms in adolescents with NSSI-D. Family upbringing factors have a great impact on NSSI-D (14, 15). According to the risk factor models, adolescents with NSSI-D tend to be emotionally sensitive and vulnerable, with personality traits closely linked to family environment (16). However, detailed observation of children’s parenting patterns in the family is difficult, correlation analysis can help clarify how parental and family factors affect adolescents’ mental health. Parents’ education level may be a factor that affects parenting patterns (17). The income of the family and the level of family harmony (how often parents quarrel) may also affect parenting styles (14, 18, 19). Additionally, poor self-concept has been identified as a correlate of NSSI-D in adolescents (20). Therefore, analyzing the associations among parents’ educational background, income, self-concept of adolescents and family harmony may help predict adolescents’ suicide risk, which may provide a basis for establishing more statistical models in the future.

In this study, we focus on the association between non-suicidal self-injury (NSSI-D) and symptoms of depression and anxiety, as well as the influence of other sociodemographic characteristics on suicide risk among adolescents. The MINI Suicide Scale was used to assess suicide risk in adolescents with NSSI behaviors in psychiatry outpatient. The participants were divided into high-risk and non-high-risk groups according to the MINISM scores, and the associations between suicide risk and other sociodemographic factors as well as anxiety and depressive symptoms were analyzed. We used SAS and SDS to test the anxiety and depression symptoms of the adolescents tested, and we observed whether anxiety and depression symptoms in adolescents who have committed NSSI-D increase the risk of suicide. We used a self-administered questionnaire answered by parents to examine the above-mentioned educational background, income, and family harmony. Our objective is to determine whether adolescents with NSSI-D who also exhibit comorbid anxiety and depressive symptoms are at an elevated risk for suicide and how parental sociodemographic factors may contribute to this risk.

## 2 Method

### 2.1 Design and participants

This study was a descriptive, cross-sectional study with correlational intentions of questionnaires and scales administered to assess patients visiting an outpatient psychiatric clinic. Adolescents aged 12 to 18 with NSSI behaviors were invited to participate in the study were adolescents aged 12 to 18 with NSSI behaviors. These adolescents were first-time visitors to the clinic and had not received any prior treatment before the reassessment. They were assessed by professional psychiatrists as meeting the diagnostic criteria for NSSI-D as specified in DSM-5. Psychiatrist also ruled out an existing other mental disorders, such as major depressive disorder, anxiety disorder, bipolar disorder, schizophrenia, obsessive-compulsive disorder and other independent mental disorder diagnoses. The diagnostic criteria for NSSI-D according to DSM-5 are as follows:

1. engagement in NSSI on 5 or more days in the past year (Criterion A);
2. the expectation that NSSI will solve an interpersonal problem, provide relief from unpleasant thoughts and/or emotions, or induce a positive emotional state (Criterion B);
3. the experience of one or more of the following: (a) interpersonal problems or negative thoughts or emotions immediately prior to NSSI, (b) preoccupation with NSSI that is difficult to manage, or (c) frequent thoughts about NSSI (Criterion C);
4. the NSSI is not socially sanctioned or restricted to minor self-injurious behaviors (Criterion D);
5. the presence of NSSI-related clinically significant distress or interference across different domains of functioning (e.g., work, relationships; Criterion E);
6. the NSSI does not occur only in the context of psychosis, delirium, or substance use/withdrawal and is not better accounted for by another psychiatric disorder or medical condition (Criterion F) (2).

Questionnaires and validated scales were administered to visitors who provided informed consent. The study was conducted at the Affiliated Hospital of Qingdao University Psychiatry outpatient clinic. Validated scales were used to measure participants’ suicide risk, level of depressive symptoms, level of anxiety symptoms and level of self-concept respectively. A self-administered questionnaire was used to investigate the parental income, educational background, and the frequency of arguments within the family. A sample of 112 participants was constructed for this study. Both adolescents and their parents signed informed consent forms. This study was approved by the Ethics Committee of Qingdao University Affiliated Hospital, and the ethics number is QYFY WZLL 28274.

### 2.2 Measurement

The MINISM (Sheehan et al., 1998) was chosen to assess the suicide risk of adolescents. The MINISM covers several topics related to suicide, including suicidal ideation, suicidal attempts, suicidal plans, and suicidal behaviors to evaluate the suicide risk of patients in the last month. The total score ranges from 0 to 52. The classification criteria of the MINISM are: Index scores 0 absence of suicide risk; 1-8 low suicide risk; 9-16 moderate suicide risk; and 17 and over high suicide risk. We defined two suicide risk categories in this study, high suicide risk(17 points or over according to MINISM) and non-high suicide risk(less than 17 points according to MINISM).

Based on previous research, many adolescents with NSSI-D have comorbid depressive or anxiety disorders, and some adolescents with anxiety and depressive symptoms have comorbid NSSI-D. We used the Self-Rating Depression Scale(SDS) and the Self-Rating Anxiety Scale(SAS) to measure participants’ anxiety and depressive symptoms. The SDS and SAS have a total of 20 items, and the items are rated on a four-point scale. The scoring method is Standard Score = Score * 1.25 with a total score of 100. The degree of depressive symptoms is expressed as the standard score/100 (21). According to the Chinese normative boundaries, symptoms were shown with the following four levels of severity ratings: Index scores 25–52 Normal; 53–62 Mild to Moderate; 63–72 Moderate to Severe; and 73 and over Severe. SAS and SDS have a similar number of entries and scoring methods. According to the Chinese normative boundaries, symptoms were shown with the following four levels of severity ratings: Index scores 25–49 Normal; 50–59 Mild to Moderate; 60-69 Moderate to Severe; and 69 and over Severe (21). The Piers-Harris Children’s Self-Concept Scale(PHCSS) is a self-report test measuring children’s self-concept. It has 60 items, and each item requires a “yes” or “no” answer and denotes one of the six domains: Behavioral Adjustment, Intellectual and School Status, Physical Appearance and Attributes, Freedom from Anxiety, Popularity, Happiness and Satisfaction. A total score of greater than or equal to 52 is defined as normal self-concept and less than 52 is defined as low self-concept (22).

We used a paper questionnaire developed by the researchers to ask adolescents’ parents to answer. The questionnaire includes an income section, a section on parents’ education level, a section on the frequency of parental arguments, a section on demographic information. The income section contained four options(RMB): less than RMB100,000, RMB100,000-RMB250,000, RMB250,000-RMB500,000, and over RMB500,000. The section on parental education contains three options: primary education level(Chinese nine-year compulsory education), secondary education level(high school education, professional learning, industrial official), and college/university or over level. The section on the frequency of parental quarrels contained five options: divorced, no quarrels, 1-2 times a month, 1-2 times a week, 3-4 times a week or more. The section on demographic information asked for age and gender of the participants’ adolescents.

### 2.3 Statistics

#### 2.3.1 Correlation analysis

The age in the sample does not follow a normal distribution, so the quartile table is used to characterize the distribution of age. For categorical variables, the researchers described the frequency of each subcategory and the proportion of the total number of participants. For multi-categorical mutually exclusive variables, we use the “one-hot” encoding technique (23). For dichotomous variables, we use “0” and “1” to represent the two categories. Based on the above principles, the level of depressive symptoms, level of anxiety, level of parental education, level of parental income, and frequency of parental arguments were coded using the “one-hot” encoding technique; gender, self-concept, suicide risk using dichotomous codes “0” and “1”.

The association was tested between level of depressive symptoms, level of anxiety and suicide risk. Logistics regression was used to examine the sociodemographic correlates of the factors. The factors of interest in this study were parental education level, income level and frequency of arguments. Next, we will find out whether educational background, income level and frequency of arguments increase the risk of anxiety and depressive symptoms in NSSI-D adolescents by the rank correlation test. The Kendall rank correlation test was used to test for correlation (24). The relationship between the strength of association and the correlation coefficient is as follows: 0.8-1.0 very strong correlation, 0.6-0.8 strong correlation, 0.4-0.6 moderate correlation, 0.2-0.4 weak correlation, and 0-0.2 no correlation. Because NSSI-D in adolescents is associated with age and gender (25, 26), with peak incidence usually at 15 years of age and a higher incidence in females than in males, risk factor investigations were adjusted for the confounding effects of age and gender.

#### 2.3.2 Network analysis

For the grading of the severity of symptoms assessed by the scales, we performed network analysis to observe the correlation among the variables (27). The degree centrality and betweenness centrality of each node in the network are calculated and sorted from high to low. All statistical analysis in this study were run by SPSS 26.0 and R 4.3.2.

First we constructed a correlation matrix $C_{n*n}$ for each variable of the unique heat coding. The algorithm for the pearson correlation coefficient for each element of the matrix is


(1) $$r_{ij}=\frac{cov\left(X_{i},\;Y_{j}\right)}{\sigma_{X}\sigma_{Y}}$$

where $cov\left(X_{i,}Y_{j}\right)$ is the covariance of the variables *X* and *Y*. $\sigma_{X}$ is the variable *X*’s the standard deviation. $\sigma_{Y}$ is the variable Y’s standard deviation in formula 1. In constructing the edges of the network, we do not set a threshold, so each element of the correlation matrix will form an edge. To make the visual network more intuitive, the strength of the correlation is mapped in terms of color and line thickness:


(2) $$color_{edge}=f\left(r_{ij}\right)$$

(3) $$width_{edge}=\omega \cdot \left|r_{ij}\right|$$

where the function *f* is used to convert the correlation value to the index of the color value. ω is a scaling factor, which is usually automatically adjusted by the parameter “edge.width = TRUE” in qgraph package in R 4.4.1. The arrangement of the nodes simulates the model of spring forces between objects in mechanics, which is known as the spring layout algorithm:


(4) $$F_{attraction}\left(d\right)=k\cdot \log\left(\frac{d_{0}}{d}\right)$$

(5) $$F_{repulsion}\left(d\right)=\frac{c}{d^{2}}$$

(6) $$P_{u}^{new}=P_{u}^{old}+\alpha \left(F_{attraction}\left(d\right)+F_{repulsion}\left(d\right)\right)$$

Here, *d* denotes the distance between nodes, $d_{0}$ is the initial desired distance between nodes, k is the coefficient of action, and c is the repulsion constant. The gravitational and draught forces between the points are calculated in formula 4 and 5. *a* is a scaling factor that controls the size of each update step. p represents the node position. The above steps are repeated until certain convergence conditions are satisfied, e.g., all nodes move less than a certain threshold or the maximum number of iterations is reached. The final position is determined through formula 6 after several iterations of computation. When the network is constructed, the network density is determined:


(7) $$Density=\frac{2E}{N\left(N-1\right)}$$

where *E* is the number of edges and *N* is the number of nodes. The downstream task after constructing the network is the analysis of node centrality. In order to highlight the edges formed between strongly correlated nodes, we performed threshold filtering for the correlation matrix:


(8) $$EDGE_{ij}=\{\begin{array}{c} 1\;if\left|r_{ij}\right|>0.2\\ 0\;\;\;\;\;else \end{array}$$

The existence of an edge is computed through formula 8 and if the result is 1 then the edge exists and if the result is 0 then the edge does not exist. After filtering the correlation matrix, the network was reconstructed following formulas 1-7, which was constituted by strongly correlated nodes. We further compute degree centrality and betweenness centrality:


(9) $$DC_{n}=k_{n}$$

(10) $$BC_{n}=\sum_{s\neq n\neq t}^{n}\frac{\sigma_{st}^{n}}{\sigma_{st}}$$

$k_{n}$ refers to the number of lines connecting each node to other nodes. Degree centrality is calculated through formula 9. $BC_{n}$ is the meso-centrality of node n. $\sigma_{st}$ is the number of shortest paths from node s to node *t* (without n). $\sigma_{st}^{n}$ is the number of shortest paths from *s* to *t* through node *v*. The number of shortest paths through the node is used to carve the Betweenness centrality and is calculated in formula 10.

#### 2.3.3 Sample size calculation and efficacy analysis

We set a threshold of 0.2 for the correlation coefficient before proceeding with the downstream task of calculating degree centrality and meso centrality. Thus the initial correlation matrix was mapped as an adjacency matrix, based on the characteristics of the new adjacency matrix, the network density was computed following formula 7, and based on the network density of this true model as well as the number of nodes, the true model was constructed in the powerly package (28). Similarly, we considered sensitivity as a performance metric with function g(γ) as the statistic.


(11) $$SEN=\frac{TP}{TP+FN}$$

(12) $$g\left(\gamma \right)=\frac{1}{R}\sum_{r=1}^{R}\left[\gamma_{r}\geq \delta \right]$$

Where SEN stands for sensitivity, TP for true positive and FN for false negative, [·] represents the Iverson brackets (29), with $\left[\gamma_{r}\geq \delta \right]$ defined as 1 if the condition $\gamma_{r}\geq \delta$ is true and 0 otherwise. The suggested sample size is chosen to ensure g(γ)≥τ , with τ being arbitrary threshold indicating a certain probability of interest, that is, the desired value for the statistic (28). The recommended sample size was calculated with 80% of the cases set to achieve 60% sensitivity.

#### 2.3.4 Sensitivity analysis

To assess the robustness of our network analysis results, we performed a sensitivity analysis using the bootstrap method. This approach involved repeated random sampling with replacement from our dataset of 112 participants. We conducted 1000 bootstrap samples to explore the stability of various network properties. For each bootstrap sample, we calculated degree centrality and betweenness centrality. Degree centrality was computed to evaluate the number of direct connections each node possesses, while betweenness centrality was assessed to determine the extent to which each node lies on the shortest paths between other nodes in the network.

By analyzing the variation in these centrality measures across the different bootstrap samples, mean values of degree centrality and betweenness centrality were computed and visually represented.

## 3 Results

### 3.1 Description

**Figure 1:**
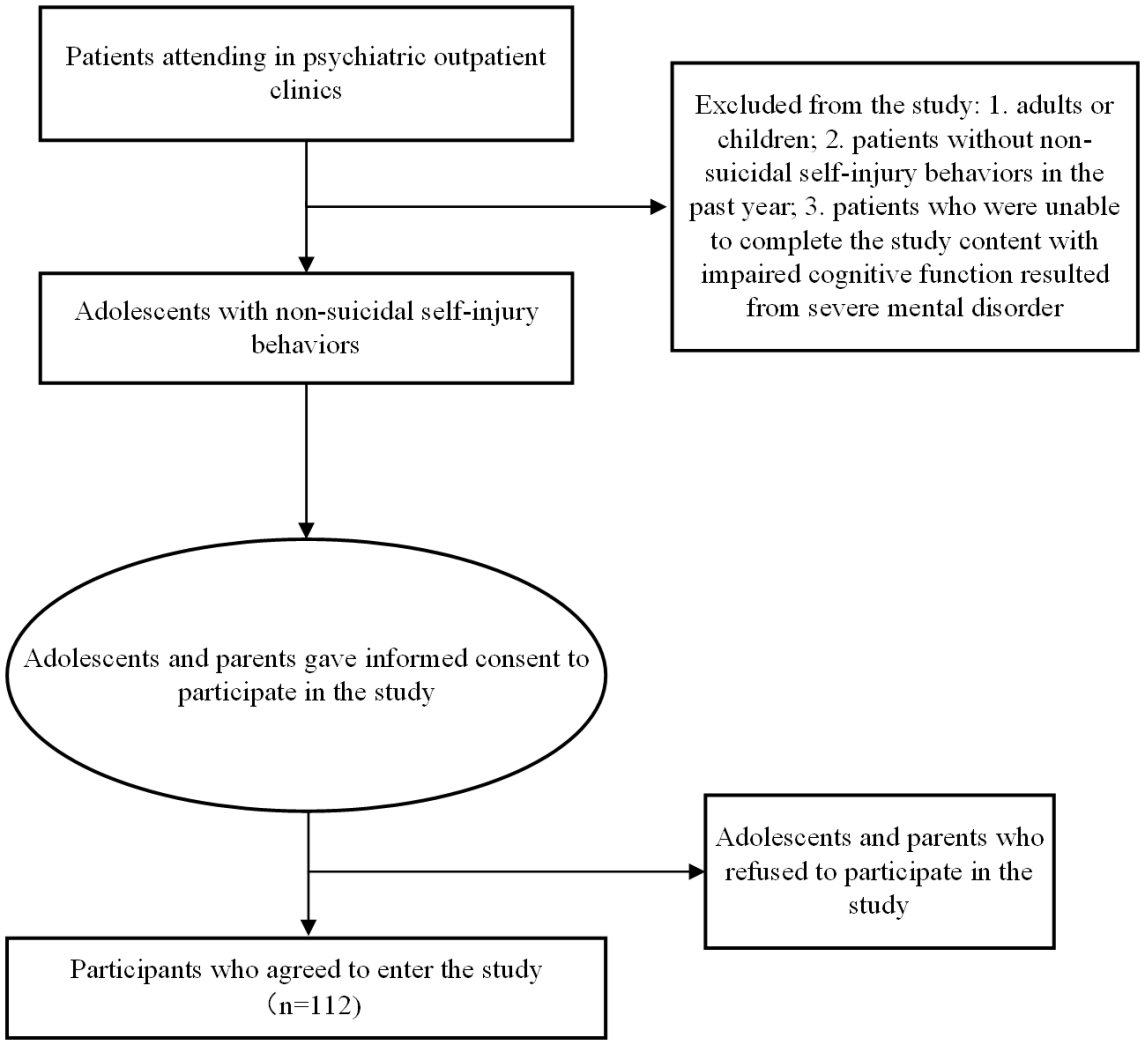
Flow chart of recruiting participants.

A total of 112 adolescent participants with concomitant NSSI-D were enrolled in this study. Participants’ survey data were completed on the day of the clinic visit. Participants did not complete all of the questions when completing the paper questionnaire and scales, with some participants completing only a portion. **Figure 1** represents the method by which participants entered this study, and **Tables 1**, **2** depict the number and missing values for each category. Because age is a continuous variable, the number of participants in each age category is described.

**Table 1:** Sociodemographic statistics.

Variable	categories	Amount(N)	Missing(N)	Rates^*^
Gender				
	Female	78	0	69.6%
	Male	34		30.4%
Father education				
	Primary education	51	0	45.5%
	Secondary education	28		25%
	College/University education	33		29.5%
Mother education				
	Primary education	54	0	48.2%
	Secondary education	28		25%
	College/University education	30		26.8%
Income				
	<10	61	0	54.5%
	10-25	35		31.3%
	25-50	12		10.7%
	>50	4		3.6%
Parents' quarrels				
	divorce	4	1	3.6%
	never	24		21.4%
	1-2 times per month	56		50%
	1-2 times per week	19		17%
	3-4 or more times per week	8		7.1%
Depression				
	No depression	14	0	12.7%
	Mild depression	14		12.7%
	Moderate depression	37		33.6%
	Severe depression	45		40.9%
Anxiety				
	No anxiety	22	2	19.6%
	Mild anxiety	28		25%
	Moderate anxiety	30		26.8%
	Severe anxiety	30		26.8%
Self-concept^b^				
	low	101	2	90.2%
	normal	9		8.0%
Suicide risk^c^		47		
	Non-high risk	64	1	57.1%
	High risk	47	42.0%

a, The rates in this column are all calculated from the number of cases/112, so in variables with missing values, the rates for each category of the variable do not add up to 100 percent; b, This study was categorized into normal and low levels based on the total score of the Self-Concept Scale; c, This study was only categorized into high risk and non-high risk based on the total score of the suicide scale.

**Table 2:** Distribution of participants' age.

Quartile of age	Age(years)
25%	14.0
50%	15.5
75%	17.0

### 3.2 Main results

#### 3.2.1 Depressive and anxiety symptoms and suicide risk

Depressive and anxiety symptoms and suicide risk were categorical variables, and chi-square and significance were calculated. The effect of depressive and anxiety symptoms on suicide risk and 95% confidence intervals were also calculated for each category. We found that both Severe depressive symptoms(OR=8.205 95%CI=3.454-19.490) and Severe anxiety(OR=3.926 95%CI=1.613-9.554) significantly increased the risk of suicide. **Table 3** gives detailed effects on anxiety and depressive symptoms levels for the other categories. The risks in this table are equivalent to the effect values from a one-way logistic regression. **Table 4** presents the results of the multilevel regulation model. We found that only Severe depressive symptoms(model 3: OR=9.29, 95%CI=2.74~31.55) was independent of the increased risk of suicide. The effect of severe anxiety on suicide risk disappears after moderating Severe depressive symptoms.

**Table 3:** Association of depression and anxiety levels with high risk of suicide.

	χ^2^	OR[95%CI]	p
No depression	8.301	0.084[0.011-0.665]	0.004^*^
Mild depression	2.974	0.322[0.085-1.229]	0.085
Moderate depression	3.849	0.435[0.188-1.008]	0.050
Severe depression	25.072	8.205[3.454-19.490]	<0.001^*^
No anxiety	6.771	0.233[0.073-0.743]	0.009^*^
Mild anxiety	0.755	0.676[0.278-1.640]	0.385
Moderate anxiety	0.006	1.035[0.443-2.414]	0.937
Severe anxiety	9.660	3.926[1.613-9.554]	0.002^*^

**Table 4:** Moderating the association of severe depression and severe anxiety on suicide risk by potential confounders.

Variable	Model 1^a^		Model 2^b^		Model 3^c^	
	Effect[95%CI]	p	Effect[95%CI]	p	Effect[95%CI]	p
Severe Depression	8.03[3.23~19.53]	<0.001	9.74[3.36~28.27]	<0.001	9.29[2.74~31.55]	<0.001
Severe Anxiety	3.43[1.36~8.65]	0.009	3.81[1.27~11.43]	0.017	1.11[0.29~4.25]	0.88

a, Model 1: Adjusted logistic regression for age and gender; b, Model 2: Additional moderated parental education level, household income, and frequency of parental arguments compared to model 1; c, Model3: The Severe depression group added Severe anxiety compared to model 2, and conversely the Severe anxiety group added Severe depression compared to model 2.

#### 3.2.2 Association of sociodemographic factors with Severe depressive symptoms and Severe anxiety

A Kendall rank correlation is used for correlation between sociodemographic factor variables and Severe depressive symptoms and Severe anxiety. We describe the variables that are significantly associated with the 0.05 test threshold in **Table 5**.

**Table 5:** Variables with correlation in Kendall's rank correlation test.

	Severe depression	Severe anxiety	Self-Concept	Father College/University Education
Severe depression	1	0.487^**^	0.248^**^	0.078
Severe anxiety	0.487^**^	1	0.183	0.327^**^
Self-Concept	0.248^**^	0.183	1	-0.028
Father College/University Education	0.078	0.327^**^	-0.028	1

a, *: p<0.05; b, **: p<0.01.

**Figure 2:**
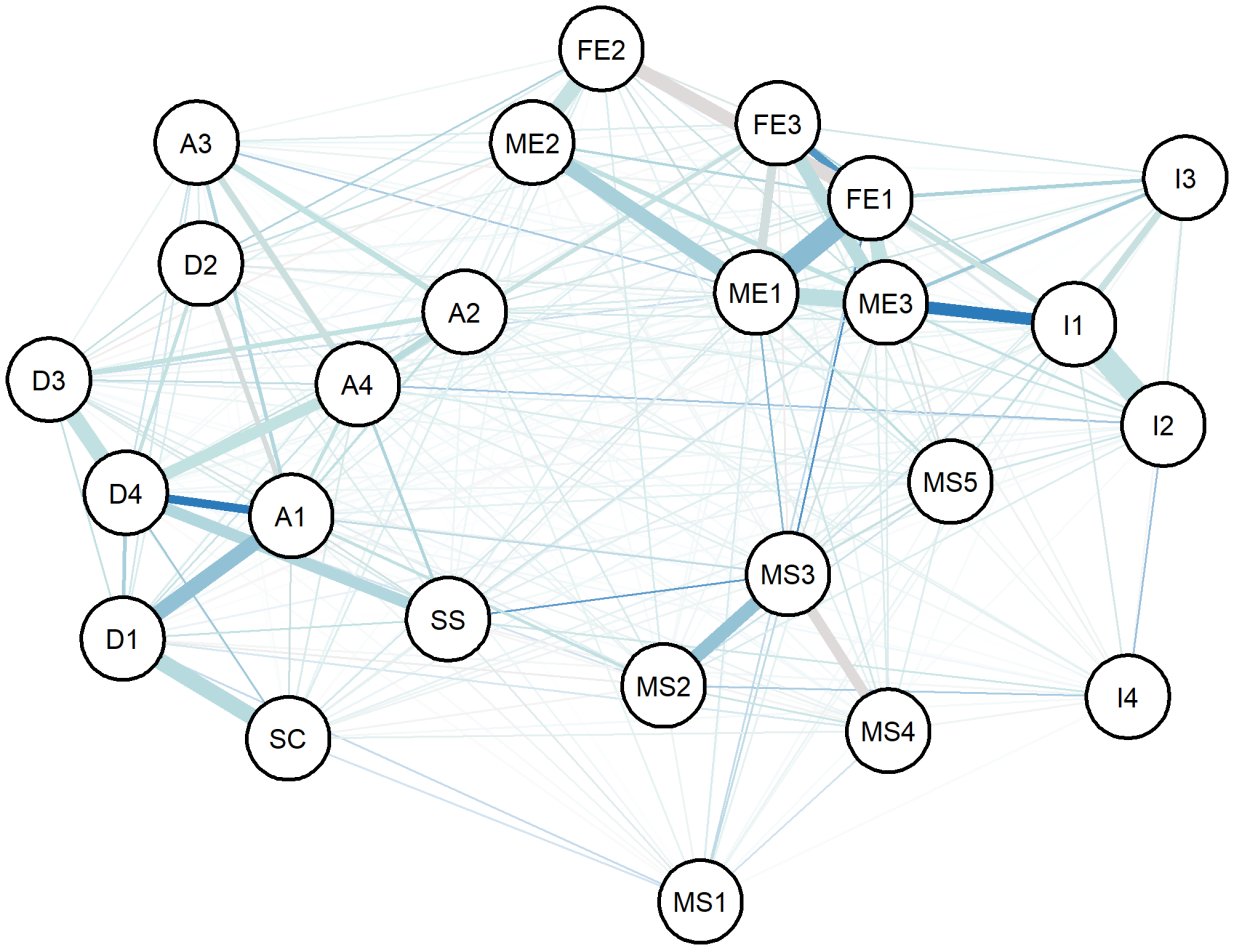
Network analysis of variables. (FE1: Father primary education, FE2: Father secondary education, FE3: Father University education, ME1: Mother primary education, ME2: Mother secondary education, ME3: Mother University education, I1: Annual Income< RMB 100000, I2: RMB 100000< Annual Income< RMB 250000, I3: RMB 250000< Annual Income< RMB 500000, I4: Annual Income > RMB 500000, MS1: divorce, MS2: never quarrel, MS3: 1-2 times quarrel per month, MS4: 1-2 times quarrel per week, MS5: 3-4 or more times quarrel per week, D1: No depression, D2: Mild depression, D3: Moderate depression, D4: Severe depression, A1: No anxiety, A2: Mild anxiety, A3: Moderate anxiety, A4: Severe anxiety, SC, Self-concept level, SS, Suicide risk).

**Figure 3:**
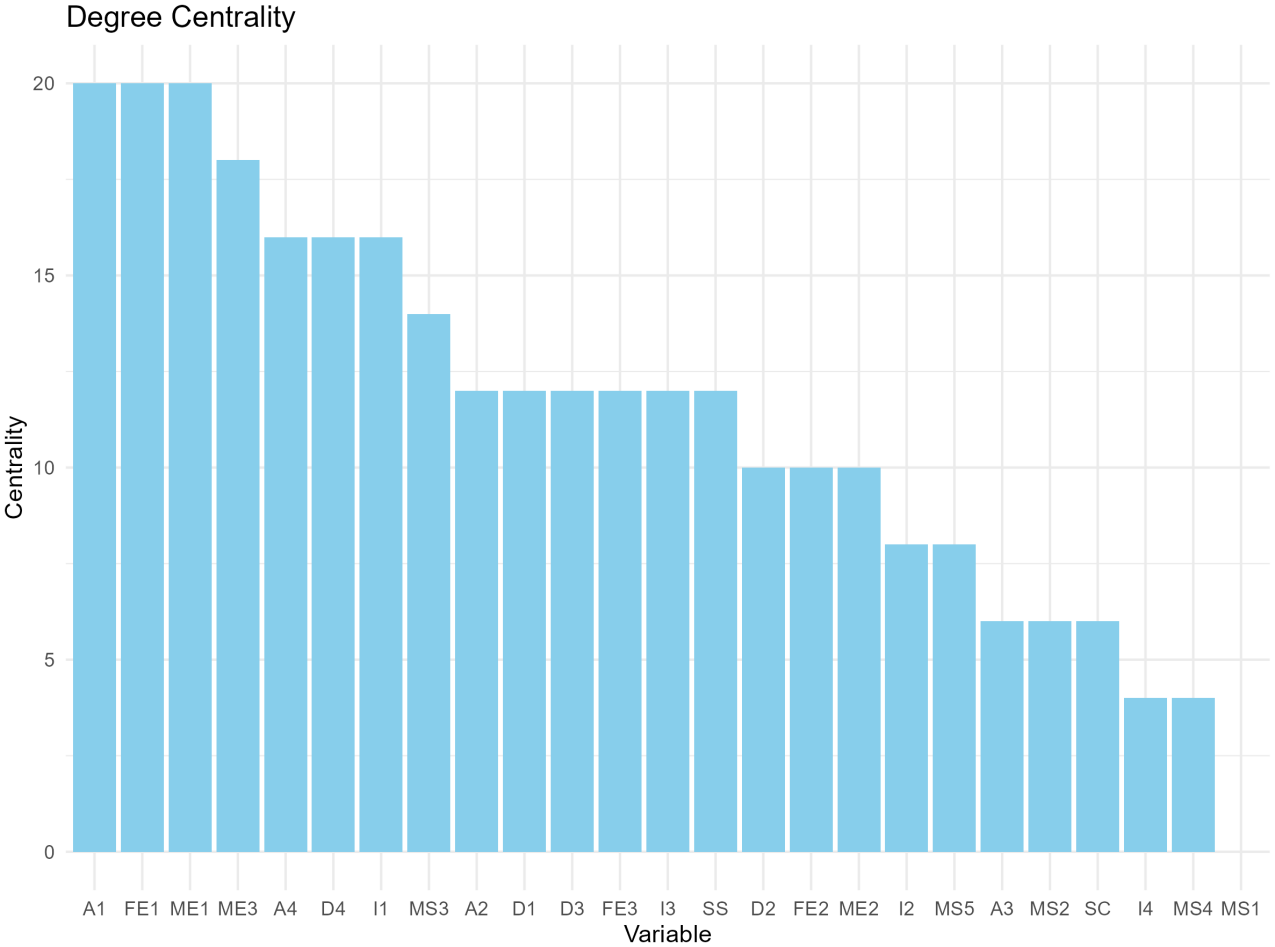
Degree centrality bar charts for 25 variables.

**Figure 4:**
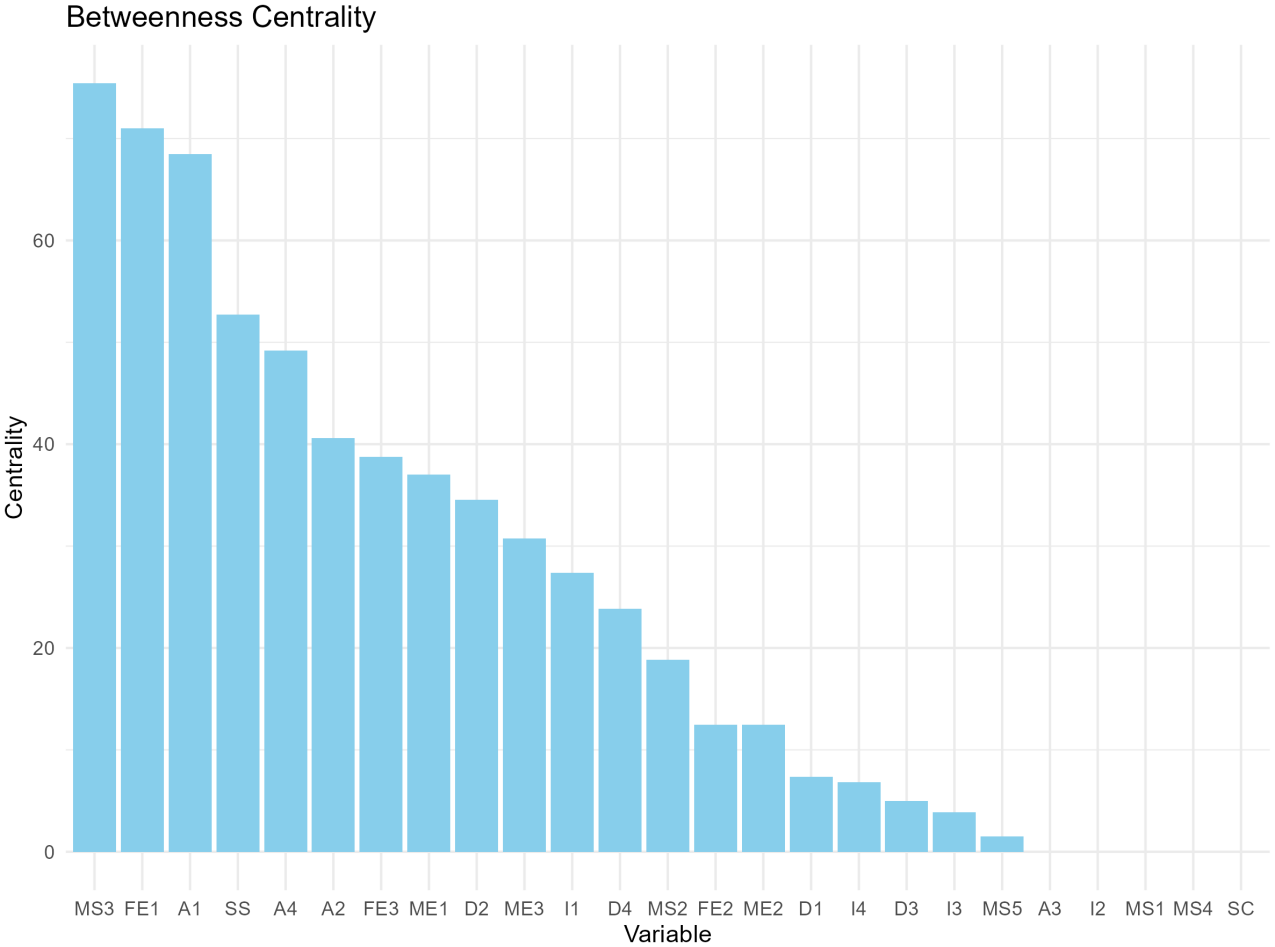
Betweenness centrality bar charts for 25 variables.

We constructed a correlation matrix for the variables of interest and created a network analysis diagram based on this correlation matrix, as shown in **Figure 2**. In the network, matrices with an absolute value of correlation coefficient greater than 0.2 were retained to calculate degree centrality and betweenness centrality. According to the degree centrality analysis, A1, FE1, and ME1 have the highest degree centrality, which means that they have the most connections with other nodes (See **Figure 3**). MS3, FE1, and A1 have the highest betweenness centrality, which may mean that they are the communication hubs of the network (See **Figure 4**).

#### 3.2.3 Recommended sample size and sensitivity analysis

**Figure 5:**
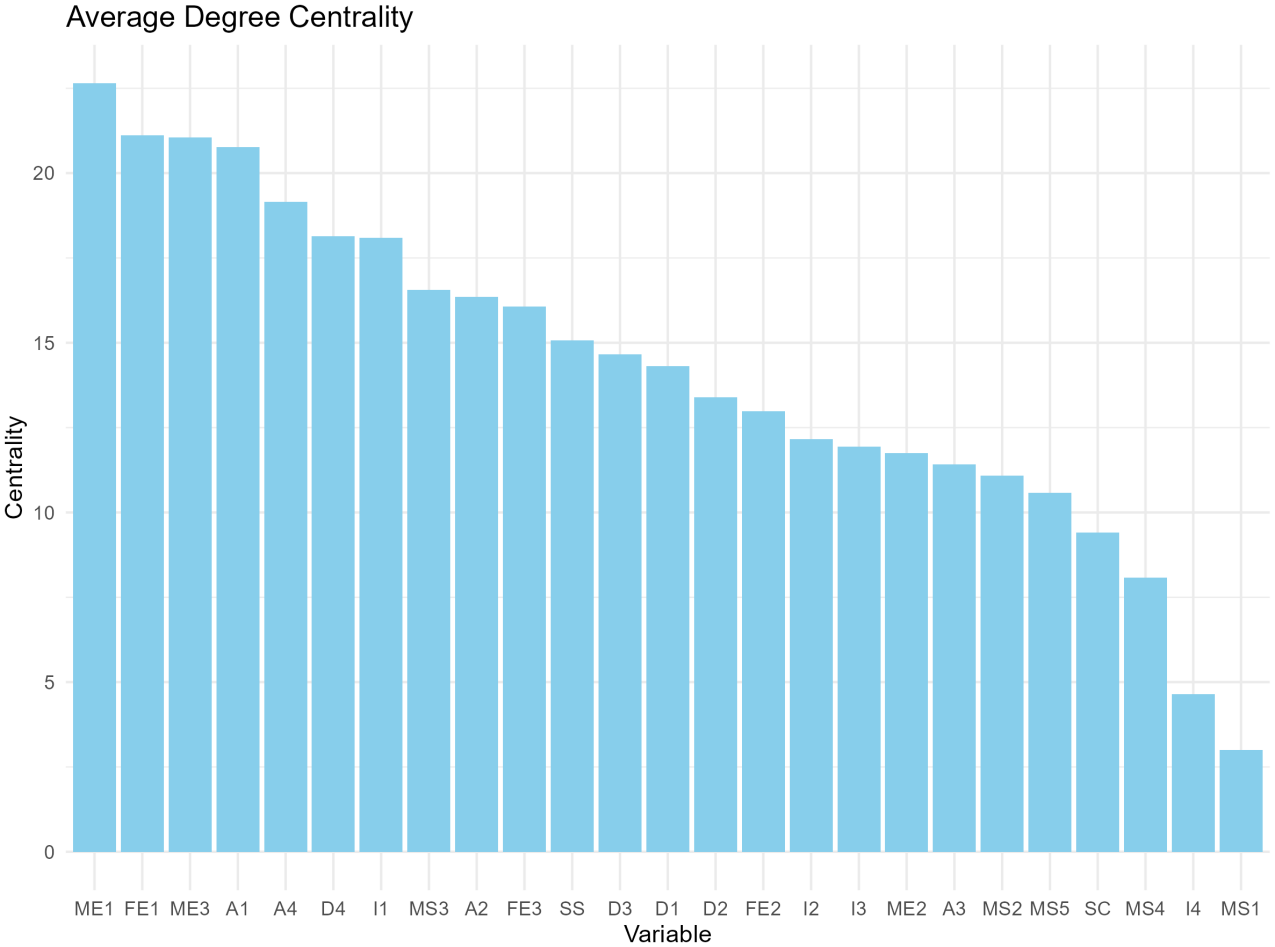
Average degree centrality based on 1000 bootstrap samples.

**Figure 6:**
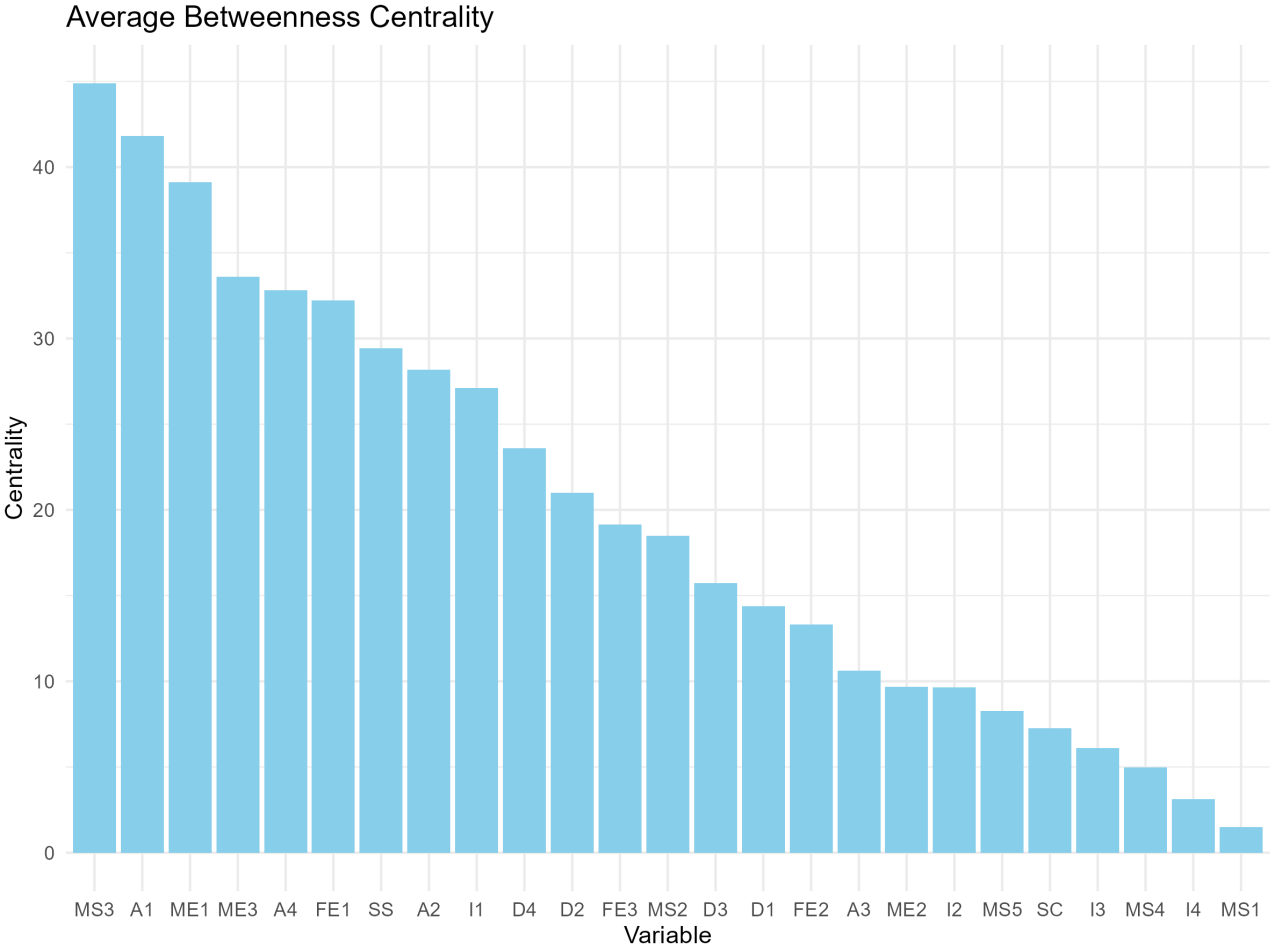
Average betweenness centrality based on 1000 bootstrap samples.

With a statistic of 0.8 and sensitivity as a performance measure of 0.6, the recommended sample size for this network model is 1000. We present the average degree centrality and average betweenness centrality of each node after 1000 bootstrap samplings (see **Figures 5**, **6**). Parental education level, anxiety level, and parental quarrel level consistently appeared as central in the network across multiple samples, indicating that these results are relatively robust. Additionally, we documented the degree and betweenness centrality results for each bootstrap sample in **Supplementary Material**.

## 4 Discussion

### 4.1 Principal finding

Results imply that adolescents with NSSI-D who exhibit severe depressive symptoms and anxiety are at a higher risk of suicide, as determined by scale evaluation and statistical analysis. Among the sociodemographic factors assessed, low self-concept was associated with severe depressive symptoms, while a father college/university education was associated with severe anxiety. Network analysis visualization shows the relationships among different factors, with color and line thickness (orange is negative correlation, and the thicker the line, the stronger the correlation). The ranking of degree centrality and betweenness centrality indicates that the father’s education level and anxiety symptoms may be key nodes, suggesting that these factors may influence various clinical manifestations and suicide risk in adolescents with NSSI-D.

### 4.2 Strengths and weakness

This study contributes to the understanding of a high suicide-risk subgroup of NSSI-D adolescents. We apply one-hot encoding based on the characteristics of the data, which can solve the problem of discrete classes of categorical data and help simplify the classification computation for machine learning (30). This study applied network analysis to address structural associations among fathers’ college/university education, self-concept, severe depressive symptoms, severe anxiety, and suicide risk.

However, several limitations should be acknowledged. Possibly due to the low sample size, we found that the frequency of severe depressive symptoms among participants with normal self-concept was 0. We were therefore unable to perform logistic regression with severe depressive symptoms treated as the dependent variable and self-concept as the independent variable, and the 0-frequency limits the application of this method, so we were unable to obtain an estimate of the association of self-concept with severe depressive symptoms when adjusting for the variables. The sample size is smaller than the recommended sample size for the expected efficacy of the network analysis, and even if the results show relative robustness, it still poses a challenge to the reliability and generalizability of the results. In addition, the odds ratio estimated by logistic regression may be higher than the actual value due to the small sample size selected in this study (31). We may have considered somewhat fewer potential confounding variables, which resulted in network analysis that did not show intertwined interactions. Finally, the cross-sectional design poses a challenge and a limitation for inferring causal relationships between factors and suicide risk in adolescents with NSSI-D.

### 4.3 Strengths and weaknesses in relation to other studies

#### 4.3.1 Negative mood and suicide risk

Previous meta-analysis have showed an association between anxiety sensitivity and suicide risk, and this association may exist in psychiatric outpatients (32, 33). Notably, Allan’s study focused on an adult population (mean age 35), whereas our study specifically targeted adolescents (32). Allan (32) pointed out that anxiety is an independent risk factor for suicide risk, a finding corroborated by numerous studies showing a strong relationship between depression and suicide risk across various populations (34–36). We believe that this association is also confirmed in the subgroup of adolescents with NSSI-D. Anxiety was not an independent risk factor for suicide risk in this study, and we hypothesize that this may be because there were more adolescents with comorbid depressive symptoms in the sample. The association between self-concept, depressive symptoms, and suicide has rarely been studied longitudinally, but the association between any two of them has been studied extensively.

#### 4.3.2 Self-concept and suicide risk

Many studies have demonstrated the mediating role of self-concept and depressive symptoms in suicide (37, 38). Self-concept reflects the self’s inner evaluation of one, low self-concept means a higher risk of self-criticism, which is associated with suicidal ideation (39). Previous researches have also demonstrated a correlation between depressive symptoms, NSSI, and self-concept (40). Additionally, the educational level of parents may affect their cognitive level, financial situation, and self-esteem level (17).

#### 4.3.3 Parental education level and suicide risk

Parents’ educational level is generally considered to be related to the way they educate their children (41). There have been many studies focusing on parental education and children’s anxiety and depressive symptoms and suicide risk. Chen conducted a meta-analysis and pointed out that the association between parental education and adolescent suicide risk is heterogeneous, and in East Asia, parental education level is positively related to adolescent suicide risk, meaning higher levels of parental education are associated with poorer mental health in offspring (42). Our findings align with these studies based on large-scale data. Generally mothers have a greater influence on their children, and the fact that mothers’ education level in the sample are lower than the fathers’ may explain the insignificant effect of mothers in the results (43). We also found a pathway in “father college/university education level-severe anxiety-severe depressive symptoms-high suicide risk”, while other levels of parents’ educational background did not show such an association. The reason may be that highly educated parents have higher expectations for their children’s performance in all aspects of life, which could increase their children’s stress and consequently their risk of mental health issues.

### 4.4 Meaning

This study shows a description of family sociodemographic factors, mood status, and suicide risk in adolescents with NSSI-D from a psychiatric outpatient setting. Network analysis revealed two main mechanistic pathways at high risk for suicide in adolescents with NSSI-D. In summary, severe depressive symptoms and severe anxiety play a central role in suicide risk, while other sociodemographic factors ultimately affect suicide risk by affecting anxiety and depressive symptoms. Adolescents with NSSI-D who exhibit with severe depressive or anxiety symptoms should be paid more attention to because they are a high-suicide risk group.

### 4.5 Further research

Future studies should focus on prospective cohort designs to better elucidate the impact of family parenting styles and parental sociodemographic factors on suicide risk in adolescents with NSSI-D. Larger sample sizes are necessary to reduce sampling error and increase the reliability of the study’s findings.

## 5 Conclusion

The results of statistical analysis suggest that severe depressive symptoms are related to the high risk of suicide (based on MINISM) in adolescents with NSSI-D statistical significantly, and anxiety symptoms and low self-concept are associated with depressive symptoms in NSSI-D adolescents. Controlling anxiety symptoms in NSSI-D adolescents may be effective in preventing them from committing suicide.

## Data Availability

The raw data supporting the conclusions of this article will be made available by the authors, without undue reservation.
